# Associations between migraine occurrence and the effect of aura, age at onset, family history, and sex: A cross-sectional study

**DOI:** 10.1371/journal.pone.0228284

**Published:** 2020-02-05

**Authors:** Yu-Wei Hsu, Chih-Sung Liang, Jiunn-Tay Lee, Hsuan-Te Chu, Meei-Shyuan Lee, Chia-Lin Tsai, Guan-Yu Lin, Yu-Kai Lin, Tsung-Han Ho, Fu-Chi Yang

**Affiliations:** 1 Department of Neurology, Tri-Service General Hospital, National Defense Medical Center, Neihu, Taipei, Taiwan; 2 Department of Psychiatry, Beitou Branch, Tri-Service General Hospital, National Defense Medical Center, Beitou, Taipei, Taiwan; 3 School of Public Health, National Defense Medical Center, Neihu, Taipei, Taiwan; Universidad de Leon, SPAIN

## Abstract

**Introduction:**

The relationships between family history, sex, age at onset, and migraine occurrence have been documented. However, the associations between these factors across different sexes and subgroups of patients have yet to be elucidated. This study evaluated the association between family history and migraine in male and female patients experiencing episodic and chronic migraine with and without aura.

**Methods:**

This cross-sectional, case–control study included 299 headache-free controls and 885 patients receiving outpatient treatment for migraine. Participants were classified into episodic (1–14 days/month) and chronic (≥15 days/month) migraine groups.

**Results:**

Positive family history was significantly more frequently observed in the episodic group than in the chronic group (49.5% vs. 26%; *P* < 0.001) in male patients, particularly in male patients without aura (50.3% vs. 21.9%; *P* = 0.003); it was less frequently observed (58.7% vs. 73.7%; *P* = 0.048) in female patients with aura. Family history was correlated with an earlier age at onset (20.7 years vs. 22.8 years; *P* = 0.002), particularly in patients without aura (21 years vs. 23.7 years; *P* = 0.002), who were women (20.9 years vs. 23.9 years; *P* = 0.002).

**Conclusions:**

Different patterns of association between family history and migraine can be observed between men and women. A positive family history of migraine is correlated with an earlier age at onset, particularly among female patients without aura.

## Introduction

Migraine is a prevalent headache disorder worldwide, affecting up to 12% of the population [1]. Over one billion people experienced migraine in 2016 [2]. The Global Burden of Diseases, Injuries, and Risk Factors (GBD) studies 2016 shows that migraine is one of the main causes of disability worldwide, particularly in young adult and middle-aged women [2]. Migraine is more frequent in women than in men and is characterized by recurrent attacks of moderate-to-severe, lateralized, throbbing, or pulsatile pain in the head. Typical episodes last from 4 to 72 hours and are often associated with photophobia, phonophobia, nausea, and vomiting; episodes may be aggravated by physical activity [3, 4]. Most people with migraine have episodic migraine. However, a subgroup of people with migraine have chronic migraine (CM), defined as at least 15 days of headache each month, including at least eight days a month on which the headache and associated symptoms are consistent with fully developed migraine attacks [4]. The prevalence of CM throughout the world ranges from 0% to 5.1%, with most general population studies reporting a prevalence of 1.4–2.2% [5]. The prevalence of CM in Asian populations ranges from 0.6% to 1.7% [6].

Similar to a number of neurological diseases, such as Parkinson’s disease [7], amyotrophic lateral sclerosis [8], and myasthenia gravis [9], migraine is not a single pathologic entity, but a syndromic disorder with many factors contributing to its clinical expression and manifestation [10]. Abnormalities involving membrane channels, receptor families, and enzyme systems, have been linked to migraine in certain groups and individuals [10]. Migraine is also a heritable disorder, in that people are at an increased risk if their first-degree families were also migraineurs; however, different familial patterns and age at onset exist in migraine with and without aura [11–13]. Few forms of migraine are linked to specific genes, whereas familial hemiplegic migraine has been linked to mutations in *CACNA1A*, *ATP1A2*, and *SCN1A* [14–16]. However, the genetic basis of many common forms of migraine remain to be elucidated [17], and epigenetic and environmental factors may also influence the presentation and age at onset of migraine [11, 18, 19]. A growing body of literature suggests that different cultural groups have different attitudes toward and meanings for pain, which may influence their neurophysiological and behavioral responses to pain [20]. The above phenomenon may be explained by cultural learning theory. Cultural learning is a unique form of social learning that allows for transmission of behaviors and information among conspecifics that is not possible in other forms of social learning [21], thereby providing the psychological basis for cultural evolution [22].

To evaluate the heritability of common forms of migraine, Genizi et al. recently investigated the association between family history and pediatric migraineurs with aura (MA). They found that these patients are more likely to have a family history of migraine than pediatric migraineurs without aura (MO) [13]. The same phenomenon was observed by Cologno et al. in adult patients [23]. In addition to this hereditary pattern, there are differences in age at onset across different groups of migraine patients, which may explain the different inheritance patterns and pathophysiological factors. Eidlitz-Markus et al. demonstrated that migraine presents at a younger age in children with a parental history of migraine, than in children with a negative family history [24, 25]. Moreover, a study showed that the age at onset in affected children was significantly lower than that in parent migraineurs [19], thus suggesting a difference in age at onset between the generations.

Although the genetic basis is still unknown, the findings of the aforementioned studies highlight the heritability of migraine, revealing differences in inheritance patterns between MA and MO and differences in age at onset between generations. However, a key limitation of these studies is the method of patient grouping, with groupings based on presentation of aura and family history. The relationships between sex, migraine frequency, and interaction effects between all these factors have not been evaluated. We hypothesize that there may be a different pattern of association between family history, age at onset, and sex in migraineurs with (MA) and without aura (MO). We hypothesized that differences may also exist across individuals who experience episodic versus chronic migraines.

The aim of the present study was to evaluate whether different familial patterns and age at onset exist between migraineurs. To this end, patients were divided into groups according to factors such as sex, frequency, and presentation of aura.

## Methods

This observational cross-sectional study adopted and met the requirements of the STROBE (Strengthening the Reporting of Observational Studies in Epidemiology) statement.

### Patients

This cross-sectional controlled study included a cohort of 1184 participants between the age of 20 and 60 years. 299 healthy, headache-free controls were volunteers recruited from the community, and 885 were participants undergoing outpatient monitoring in a headache clinic at the Department of Neurology of Tri-Service General Hospital (TSGH) between November 2015 and April 2018. The study protocol was approved by the Institutional Review Board of TSGH (TSGH-IRB-1-105-05-006), and all the participants provided informed written consent prior to enrollment. Patients suffering from migraine, both with and without aura, were enrolled. After excluding patients with concomitant primary and secondary headache disorders, those with insufficient data to determine migraine frequency, and those lacking information on clinical characteristics, a total of 885 patients were included in the final analysis.

Each participant completed a screening questionnaire and was subsequently interviewed directly by a board-certified neurologist and headache specialist (FCY); diagnoses were based on criteria defined in the International Classification of Headache Disorders, 3rd edition [4]. Thus, participants were divided into three groups: those with episodic migraine (1–14 days/month; N = 697); those with chronic migraine according to the ICHD-3 criteria, which was defined as at least 15 days of headache each month, including at least eight days a month on which the headache and associated symptoms are consistent with fully developed migraine attacks (≥15 days/month; N = 188); and headache-free controls with no history of headache disorders (N = 299). All headache-free controls were interviewed directly by the FCY, and reported no previous diagnosis of headache disorders including primary headache such as migraine, tension type headache, cluster headache, and other secondary headache. They were also examined and were found to have no history of physical, cognitive, or degenerative diseases of the central nervous system, or severe head injuries with loss of consciousness. However, there may be a small number of patients with extremely infrequent headaches, whose data may have been missing or misinterpreted in large studies.

Patients in the episodic and chronic migraine groups were further divided into four groups based on sex and the presentation of aura. Of the 885 participants with migraine, 291 (32.9%) experienced aura and 594 did not experience aura. The enrolment process is illustrated in S1 Fig.

Participants were checked for any history of physical, cognitive, or degenerative diseases of the central nervous system or severe head injuries with loss of consciousness. All participants underwent detailed neurologic examinations as well as Doppler ultrasound examination by an experienced radiologist to screen for any possible underlying vascular disorders including cervical artery dissection that might cause secondary headache and mimic migraine [26, 27]. The Doppler examination was performed within the certificated Neurovascular Ultrasound Laboratory in Tri-Service General Hospital, Taipei, Taiwan. Each participant underwent color-coded duplex ultrasonography (CCDU) of the cervical vessels with an ATL HDI 5000 ultrasound system (Philips, Bothell, WA, USA) with an L12-5 linear 38 mm transducer. Participants were diagnosed according to the laboratory’s ultrasound criteria [28, 29]. No definitive abnormalities were identified in any of the patients.

### Patient assessments

#### Evaluation of family history and age at onset

Information on the family history and age at onset of headaches was collected using a structured questionnaire. Participants were then interviewed by a board-certified neurologist and headache specialist (FCY). The presentation of migraine and accompanying symptoms was clarified through an interview. The participants’ family members who were present and reported to have migraine were also interviewed by FCY. Information regarding headache in other relatives who were not present was obtained second-hand from those who were present. Patients were assigned to the positive-family-history group only if at least one of their first-degree relatives had migraine-like headaches; other types of headache and secondary causes were excluded. The structured questionnaire comprised the Beck Depression Inventory-II [30], the Hospital Anxiety and Depression Scale [31], a restless legs syndrome (RLS) screening questionnaire from the RLS Foundation [32], the Pittsburgh Sleep Quality Index [33], and the Migraine Disability Assessment questionnaire [34], which were all proven to be both reliable and valid [30, 32, 33, 35, 36]. The relationship between these factors and migraine has been previously assessed and published.

### Data analysis

The normality of continuous variables was tested using a Kolmogorov-Smirnov test. However, none of the normality tests met the assumption due to the relatively large sample size of this study. According to the normality plot (the normal Q-Q plot) and the standard deviation values relative to means, the distribution of these continuous variables was not far from normal (data not shown). Data for continuous and categorical variables are expressed as mean ± standard deviation and frequency and proportion, respectively. Differences in continuous and categorical variables between the study groups (control, episodic, and chronic) were tested using one-way analysis of variance and chi-squared test, respectively. Pairwise comparisons were performed using the Bonferroni adjustment, only where an overall test was significant. The proportions of patients with a migraine family history in the study groups were compared using the chi-squared test and Bonferroni-adjusted multiple comparison.

Age at onset of migraine in patients with and without family history was compared using the Student *t*-test, and the analysis was further stratified by aura and sex. Finally, to investigate the association between baseline characteristics and risks of migraine, we performed a multivariable logistic regression analysis. With the assumption of a mean age at onset of 20 years in the patients with a family history of migraine and 22 years in patients without a family history of migraine, a standard deviation of 10 years, and an alpha level of 5%, a minimum sample size of 686 patients with migraine was required to achieve a power of 80%. A two-sided *P* value of <0.05 was considered to be statistically significant. No adjustment for multiple testing (multiplicity) was carried out in this study to avoid low statistical power. Statistical analyses were conducted using SPSS 22 (IBM SPSS, Armonk, NY: IBM Corp).

## Results

### Characteristics of study participants

Table 1 presents the characteristics of the participants in the control and migraine groups. The study cohort comprised 299 participants in the control group (25.3%), 697 patients in the episodic migraine group (58.9%), and 188 patients in the chronic migraine group (15.9%). The prevalence of migraine aura was higher in the chronic migraine group than in the episodic group (39.9% vs. 31%; *P* = 0.021). The proportion of smoking was higher in the two migraine groups than that in the control group (*P* = 0.015). No significant difference was observed regarding sex, age, and alcohol and coffee consumption between the three study groups. The scores for anxiety, depression, and sleep quality were worse for the chronic migraine group than for the episodic migraine and control groups (*P* < 0.001). The proportion of family history of migraine was higher in the migraine groups (51.8% and 52.1% in the episodic and chronic migraine groups) than that in the control group. No significant difference was observed between the episodic and chronic migraine groups.

**Table 1:** Prevalence of aura, demographics, substance use, migraine severity, psychometric inventories, and family history of migraine in the study population in the control, episodic migraine, and chronic migraine groups (N = 1184).

Variable	Control	The migraine group		*P* value
		Episodic (1–14 days)	Chronic (≥ 15 days)	
Patient number	299	697	188	—
Aura	—	216 (31.0)	75 (39.9)	0.021
Sex				0.374
Female	205 (68.6)	475 (68.1)	138 (73.4)	
Male	94 (31.4)	222 (31.9)	50 (26.6)	
Age (years)	35.8±12.4	35.0±11.1	35.7±13.0	0.581
Smoking	42 (14.0)	146 (20.9)^a^	44 (23.4)^a^	0.015
Alcohol drinking	91 (30.4)	265 (38.0)	63 (33.5)	0.061
Coffee consumption				0.233
Never	71 (23.7)	160 (23.0)	41 (21.8)	
< once a month	89 (29.8)	166 (23.8)	54 (28.7)	
≥ 1 day a week	139 (46.5)	371 (53.2)	93 (49.5)	
MIDAS	—	21.5±14.8	29.8±19.0	<0.001
BDI total score	7.0±6.2	9.9±7.9^a^	13.4±9.6^ab^	<0.001
HADS–anxiety	5.7±3.4	7.6±4.2^a^	9.0±4.4^ab^	<0.001
HADS–depression	4.3±3.1	5.5±4.0^a^	6.9±4.3^ab^	<0.001
PSQI total score	7.2±3.3	8.6±3.7^a^	10.6±4.2^ab^	<0.001
Family history of migraine	94 (31.4)	361 (51.8)^a^	98 (52.1)^a^	<0.001

MIDAS, Migraine Disability Assessment; BDI, Beck Depression Inventory; HADS, Hospital Anxiety and Depression Scale; PSQI, Pittsburgh Sleep Quality Index;

“**a**” indicates significant difference between the migraine and control groups on Bonferroni multiple comparison;

“**b**” indicates significant difference between the control and episodic migraine group on Bonferroni multiple comparison;

Continuous data are presented as mean ± standard deviation and categorical data are expressed as frequency and percentage.

### Family history of migraine in control and migraine groups

**Fig 1:**
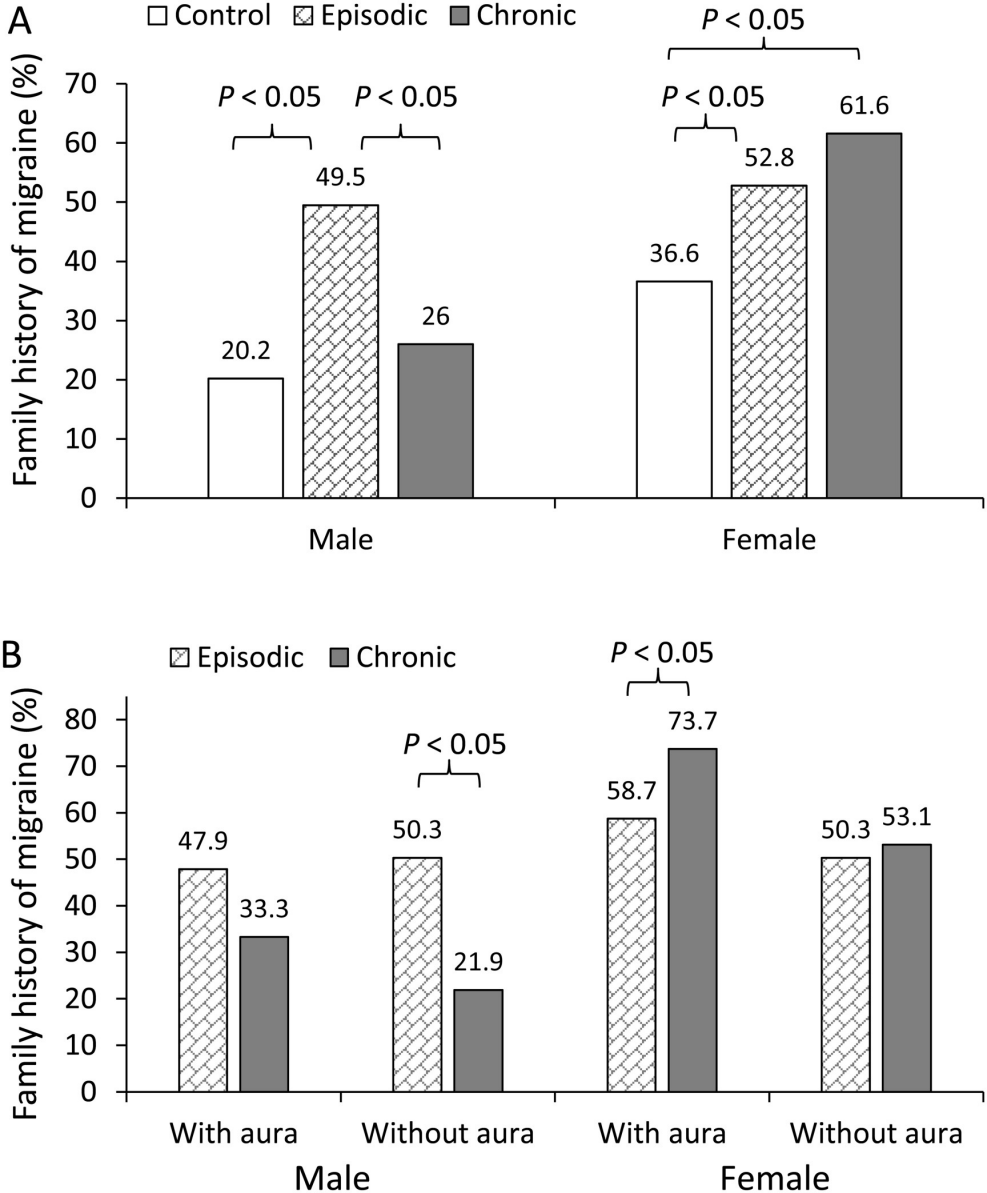
Family history of migraine in control and migraine frequency groups stratified by sex (A) and in episodic and chronic migraine groups stratified by sex and aura (B). In the male participants, the proportion of individuals with a family history of migraine was significantly lower in the chronic migraine group than in the episodic migraine group (26% vs. 49.5%; P < 0.05). However, this effect was not observed in the female participants (Fig 1A). In the subgroup of male patients without aura, the proportion of individuals with a family history of migraine was lower in the chronic migraine group than in the episodic migraine group (21.9% vs. 50.3%; P = 0.003). However, in female patients with aura, the proportion of individuals with a family history of migraine was more frequent in the chronic migraine group than in the episodic migraine group (73.7% vs. 58.7%; P = 0.048; Fig 1B).

Table 2 shows the family history of migraine in the control and migraine groups, as stratified by aura and sex. No significant differences between the episodic and chronic migraine groups were observed in the full sample or in the female subgroup. However, in the male participants, the proportion of family history was significantly lower in the chronic migraine group than in the episodic migraine group (26% vs. 49.5%; *P* < 0.05; Fig 1A). Further analysis based on stratification by aura and sex revealed that in the subgroup of male patients without aura, the proportion of family history was lower in the chronic migraine group than in the episodic migraine group (21.9% vs. 50.3%; *P* = 0.003). However, in female patients with aura, the proportion of family history was more frequent in the chronic migraine group than in the episodic migraine group (73.7% vs. 58.7%; *P* = 0.048; Fig 1B).

**Table 2:** Family history of migraine in controls and in patients with migraine stratified by sex and aura. The upper part was the total number of subjects and those stratified by sex. The middle part was the subjects with aura and those stratified by sex. The lower part was the subjects without aura and those stratified by sex.

Subgroup	Control	Episodic (1–14 days)	Chronic (≥ 15 days)	*P* value
Total				
Total	94 (31.4)	361 (51.8)^a^	98 (52.1)^a^	<0.001
Male	19 (20.2)	110 (49.5)^a^	13 (26.0)^**b**^	<0.001
Female	75 (36.6)	251 (52.8)^a^	85 (61.6)^a^	<0.001
With aura				
Total	—	119 (55.1)	48 (64.0)	0.179
Male	—	35 (47.9)	6 (33.3)	0.264
Female	—	84 (58.7)	42 (73.7)	0.048
Without aura				
Total	—	242 (50.3)	50 (44.2)	0.246
Male	—	75 (50.3)	7 (21.9)	0.003
Female	—	167 (50.3)	43 (53.1)	0.653

Data are expressed as frequency and percentage;

“**a**” indicates significant difference versus the control group in the Bonferroni multiple comparison;

“**b**” indicates significant difference versus the episodic group in the Bonferroni multiple comparison.

### Age at onset of migraine

**Fig 2:**
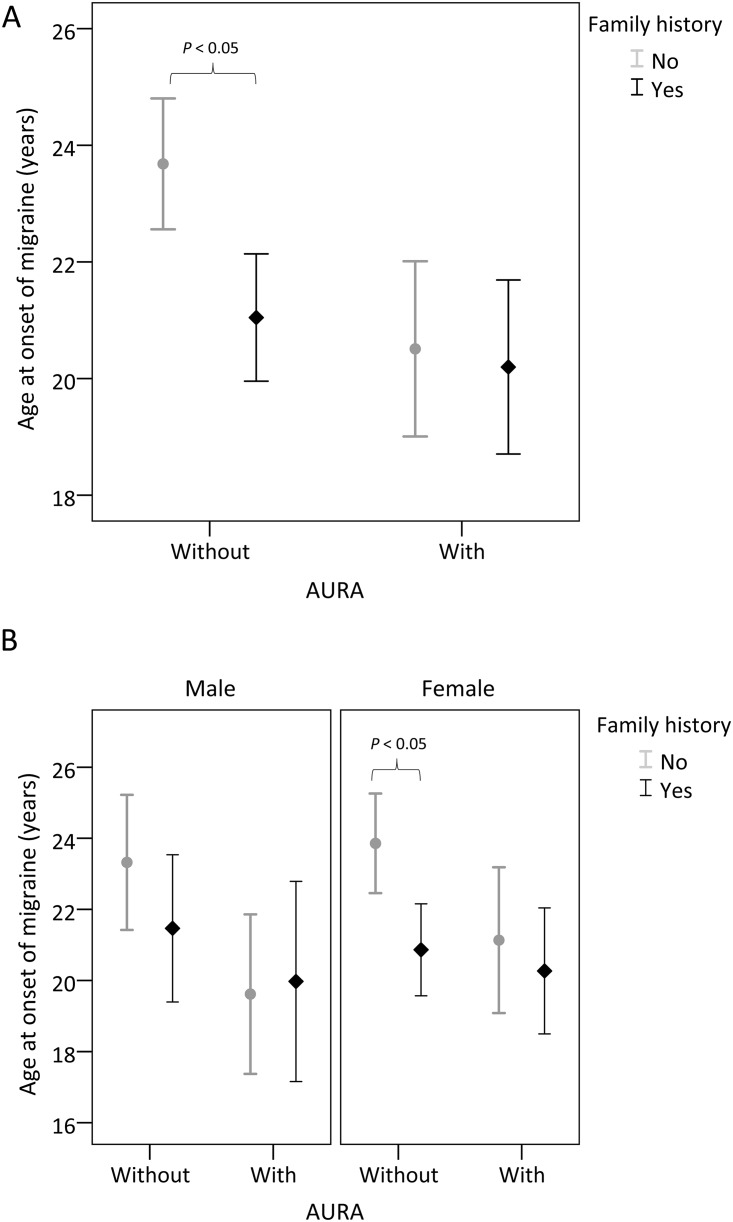
Age at onset in patients with and without a family history of migraine, as stratified by aura (A) and sex (B). The middle horizontal line represents the mean, and the error bar represents the 95% confidence interval around the mean values. The age at onset was earlier in patients with a family history than in those without (20.7 vs. 22.8 years; P = 0.002); however, this phenomenon was observed only in patients without aura (Fig 2A). The familial difference in age at onset was present only in female patients without aura (20.9 vs. 23.9 years; P = 0.002) but not in male patients without aura (Fig 2B).

Table 3 presents the age at onset of migraine in patients with and without family history. The result showed that the age at onset was earlier in patients with a family history than in those without (20.7 years vs. 22.8 years; *P* = 0.002); however, this phenomenon was observed only in patients without aura (Fig 2A). Further analysis through stratification by aura and sex showed that the familial difference in age at onset was present only in female patients without aura (20.9 years vs. 23.9 years; *P* = 0.002) but not in male patients without aura (Fig 2B).

**Table 3:** Age at onset of migraine in patients with and without family history stratified by aura and sex. The upper part was the total subjects and those stratified by sex. The middle part was the subjects with aura and those stratified by sex. The lower part was the subjects without aura and those stratified by sex.

Subgroup	With family history	Without family history	*P* value
Total			
Total	20.7±9.0	22.8±9.2	0.002
Male	21.0±8.9	22.1±8.8	0.313
Female	20.6±9.1	23.1±9.4	0.002
With aura			
Total	20.2±9.3	20.5±8.1	0.776
Male	20.0±8.4	19.6±7.6	0.840
Female	20.3±9.6	21.1±8.4	0.541
Without aura			
Total	21.0±8.8	23.7±9.4	0.001
Male	21.5±9.1	23.4±9.1	0.168
Female	20.9±8.7	23.9±9.6	0.002

Data are presented as mean ± standard deviation.

### Factors associated with the risk of migraine

Table 4 displays the association between baseline characteristics and risks of migraine. The results demonstrated that the presence of family history (adjusted odds ratio [aOR] 2.10, 95% confidence interval [CI] 1.56–2.81), smoking (aOR 1.75, 95% CI 1.15–2.65), higher anxiety score (aOR 1.09, 95% CI 1.03–1.14) and poor sleep quality (aOR 1.08, 95% CI 1.04–1.13) were significantly associated with higher risks of migraine.

**Table 4:** Multivariable logistic regression analysis of factors associated with the risk of migraine.

Variable	aOR (95% CI)	*P* value
Family history	2.10 (1.56–2.81)	<0.001
Age (years)	0.99 (0.98–1.00)	0.172
Female sex	1.01 (0.72–1.41)	0.952
Smoking	1.75 (1.15–2.65)	0.009
Alcohol drinking	1.10 (0.79–1.52)	0.569
Coffee consumption		
Never	Reference	—
< once a month	0.82 (0.55–1.22)	0.329
≥ 1 day a week	1.10 (0.76–1.58)	0.607
BDI total score	1.02 (0.99–1.05)	0.285
HADS–anxiety	1.09 (1.03–1.14)	0.001
PSQI total score	1.08 (1.04–1.13)	0.001

aOR, adjusted odds ratio; CI, confidence interval; BDI, Beck depression inventor; HADS, hospital anxiety and depression scale; PSQI, Pittsburgh sleep quality index;

HADS–depression was omitted in the model because its multicollinearity with BDI total score.

## Discussion

In the present study, patients with migraine had a higher proportion of first-degree relatives with migraine than the controls; nevertheless, no significant differences were observed between the episodic and chronic migraine groups. However, after stratifying by sex, a difference in family history and aura presentation emerged in that the prevalence of a positive family history was higher in male patients without aura in the episodic group. Conversely, the prevalence of a positive family history was higher in female patients with aura in the chronic migraine group. Based on similar stratification strategy, we also evaluated the age at onset of migraine and categorized the participants into groups based on sex, presentation of aura, and family history. We observed that the age at onset of migraine was earlier in patients with family history than in those without (20.7 years vs. 22.8 years; *P* = 0.002). The age of onset in male participants was 21 years and 22.1 years for those with and without a family history of migraine, respectively, and was not statistically significant (*P* = 0.313). In contrast, the age of onset in female participants was 20.6 years and 23.1 years for those with and without a family history of migraine, respectively, and was statistically significant (*P* = 0.002). The age difference was more clinically significant in females (2.5 years) than in males (1.1 years). After further stratification by aura and sex, this phenomenon was observed only in patients without aura and female patients without aura.

Although controversial, previous studies have suggested a higher risk of migraine in first-degree relatives of both MA and MO [12, 23]. Our results show the same family-history pattern in female patients with aura in the chronic migraine group. Previous studies have found that first-degree relatives of MO have increased risks of both migraine without and with aura [37, 38]. In our study, we demonstrated that in male patients without aura, first-degree relatives had a higher risk of headaches. Therefore, the results of the present study, consistent with those of previous studies, indicate that migraine is a heritable disorder in both MO and MA. Moreover, the results of this study show that family history was significantly lower in the male chronic migraine group than in the male episodic migraine group and in the female group. It has been reported that chronic daily headache carries a substantial genetic predisposition [39]. Recent studies have also suggested that women are more susceptible to developing chronic migraine [5, 40]. A family history of migraine was found to be significantly more prevalent in the chronic migraine than in the episodic migraine group [40]. However, it remains difficult to conclude that men are more susceptible to developing chronic migraine than women, in whom there is no apparent family history suggesting a genetic predisposition. This finding should be assessed in further studies.

In the evaluation of the age at onset of migraine in different groups, although limited information exists regarding the age at onset of migraine in different types and in sexes, our results are also consistent with those of previous studies showing early onset ages in patients with a positive family history [24, 25].

By stratifying the participants into different groups by sex, migraine frequency, and presentation of aura, we observed different patterns of association between these groups and thus delineated the hereditary patterns of migraine. Our results suggest that sex may be an important factor influencing the heritability, frequency, and presentation of migraine aura; nevertheless, the underlying mechanisms are currently unknown. Although, family history was associated with a higher risk of headache in the migraine groups than in the control group, the risk was especially predominant in male patients without aura in the episodic migraine group, and in female patients with aura in the chronic migraine group. Moreover, regarding the influence of sex and aura on the age at onset of migraine, our study highlighted a difference in the age at onset between men and women and between the patients without aura and those with aura. Previous research could not detect these differences, likely because of the relatively small sample size compared with that in the present study [24].

Many previous studies have reported the association between genetic, epigenetic, and environmental factors and the presentation of migraine [11, 18, 19]. In this study, we also observed that the presence of family history, smoking, higher anxiety score, and poor sleep quality were significantly associated with higher risks of migraine. The relationship between anxiety, sleep quality, and migraine has been previously assessed and published [41, 42]. Moreover, in our present study, differences in family history associated with both sex and migraine were observed. These differences suggest that different pathophysiological factors and mechanisms involved in the formation of migraine may exist between the sexes. For example, due to fluctuations of estrogen levels in women, sex hormones, the menstrual cycle, pregnancy, the use of hormonal contraceptives, and menopause modulate the attack frequencies of migraine [43]. The influence of family history on the age at onset was also observed in the current study. In addition to genetic factors, this difference may be due to the influence of parental behavior on children’s pain experiences [44–46] because children of migraineurs may experience their parents’ reaction to pain and learn to complain of pain earlier, thus presenting an earlier age at onset of migraine than do migraineurs without family history [19, 44–46]. This study did not directly assess the pain of migraine and headache, and this could be an avenue for future research.

Chronic or recurrent pain is a common complaint of childhood, and the most common condition is headache [47]. It can arise from many physical health conditions or emerge idiopathically [47]. Social learning and family educational factors have been proven to be influential forces in shaping how children respond to pain [48]. Parental verbalization and non-verbal behavior may function as a signal for their concern and precipitate children’s behavioral distress and may serve to reinforce children’s pain as the interaction progresses [44]. However, this effect may vary between genders. Chambers et al. have demonstrated that maternal behavior has a direct impact on the daughters’ subjective reports to pain and hypothesized that girls may be more sensitive to their parents’ behavior regarding pain symptoms [44]. The results of our study, consistent with those of previous studies, revealed a different pattern of association between family history and migraine in men and women.

In the treatment of migraine, multidisciplinary headache management that includes pharmacological, non-pharmacological, and educational approaches has been considered the best form of treatment [49]. Biobehavioral treatments for patients with migraine should include therapeutic patient education (TPE) and self-care, cognitive behavioral interventions, and biobehavioral training. A recent study revealed strong evidence for intermediate-term disability improvement and decreased headache frequency after TPE in adult patients with migraines [49]. It also revealed that TPE might improve the quality of life in the intermediate term. Therefore, it is important for the TPE approach to be part of a comprehensive intervention offered to patients with migraine.

Although this study involved a well-controlled design, demographically homogeneous groups, and analysis of subgroups by different sexes, frequencies, and presentation of aura, some limitations exist. First, the prevalence of family history and the age of onset were established based on a structured questionnaire and subsequent interviews. Episodes of migraine in children may have a shorter duration of attack with excellent recovery within a short duration and there is the possibility of bilaterally located pain. Moreover, the frequency or attacks in pre-pubertal patients could be extremely low [50, 51], and the symptoms associated with headache, such as photophobia and phonophobia, are rarely mentioned by the young patient [50]. Thus, migraine in children may be difficult to diagnose and easily missed in an interview. Therefore, the age at onset may be a potential confounder. In this study, children were counted as primary family members and every participant was questioned about migraine in their children. However, in the present study, the age of these children was not recorded; moreover, they may not have experienced migraine yet. We will take this into consideration in our future study and record the children’s age; thus, we can continue to observe whether they will develop migraine and exclude these confounding factors. Moreover, the children may have migraine equivalents before developing migraine. Because this was not a prospective study, the interview with the participants may have been subject to recall bias. Second, the relationship between the participants and their first-degree relatives with migraine, whether paternal or maternal, was not recorded, and information about headache in the grandparents and other relatives was obtained second-hand. All the participants were interviewed about their occasional episodes of migraine and other types of headache in their primary family members. However, there may be an extremely small number of primary family members with infrequent headaches, whose data may be missing or misinterpreted in large studies because they did not reveal their migraine history due to personal reasons or due to lack of observation by the participants. The interview with the family members with very infrequent headaches may also have been subject to recall bias. In future, we will take this into consideration and exclude these confounding factors. Third, recent studies have suggested that migraine with aura is associated with an increased risk of carotid thickening, whereas migraine without aura is associated with a low risk of carotid plaques and arterial stiffening [52]. In the present study, we did not evaluate the relationship between migraine and the markers of vascular damage. The relationship between these factors will be examined and clarified in future studies. Finally, our study deals only with an Asian population from Taiwan, and the study group was restricted to patients attending the Tri-service General Hospital outpatient department. The study group may have more severe disease than that observed in community-derived patients. Our data might also reflect referral patterns and other idiosyncrasies of our clinic and of the Asian population from Taiwan, such as the physician’s decision to refer the patient or the patient’s agreement to attend our clinic. Some patients may be less aware of milder, less-frequent headache, leading to a lower admission rate to our clinic. In younger patients, parents with a history of migraine may have early recognition in the same disease as their children, thereby limiting the broad generalizability of the findings [19]. Therefore, our conclusions cannot be extrapolated to the general population with migraine. Additional population-based, multinational, multicenter studies with larger sample sizes are warranted to corroborate our findings.

In a previous study conducted by Russell and Olesen, visual aura was identified as the most frequent manifestation, and was present in 99% of participants experience migraine with aura, followed by sensory (31%), aphasic (18%), and motor (6%) symptoms [53]. This study suggests that the use of visual phenomena as a marker for aura should identify patients having migraine with aura with a high probability. However, patients experiencing migraine with aura may not have aura with every headache. In a previous study, in patients who reported aura, the average percentage of occurrence of aura with headache was 19.7% [54]. This study also suggested that 79% of patients having migraine with aura had both migraine with aura (MA) and migraine without aura (MO) [54]. In our study, because symptoms such as confusion, dysphasia, paresis, and sensory loss can be difficult to evaluate as aura, we only included migraineurs with visual aura in the MA group. We collected the information on aura with a structured questionnaire, and the characteristics of aura were subsequently clarified through a direct interview. In our study, the diagnosis of migraine with aura was based on the criteria defined by the International Classification of Headache Disorders, 3rd edition (ICHD-3) [4], and the participants were questioned carefully about the presentation of aura. In participants with infrequent aura, the diagnosis was based on the criteria of ICHD-3. However, there may be a small number of patients with very infrequent aura mixed with MO in large studies. Moreover, we did not enroll patients with migraine aura without headache in this study; details of the migraine, such as the frequency of attack and the presentation of aura in the first-degree relatives of the participants, were not available. In future, we will take this into consideration. Future studies should include other types of aura with more detailed information of the presentation of migraine; the paternal or maternal relationship may aid researchers to understand the hereditary pattern of migraine.

## Conclusion

In conclusion, the results of the present study reveal a different pattern of association between family history and migraine in men and women. Moreover, the results demonstrate that a positive family history correlates with an earlier age at onset, particularly among female patients without aura. Thus, this study highlights the associations between migraine prevalence, aura, age at onset, family history, and sex. Future studies should investigate these factors in more detail, clarifying the effects of paternal versus maternal relationships and how different forms of migraine in first-degree relatives influence migraine risk.

## Supporting information

S1 Fig (TIF)

S1 Data (XLSX)

S1 Checklist (DOC)

## Abbreviations

MA: migraine with aura
MO: migraine without aura
RLS: restless legs syndrome
TSGH: Tri-Service General Hospital
