# Supplementary figures and images for: Associations between migraine occurrence and the effect of aura, age at onset, family history, and sex: A cross-sectional study
Source: PLoS One. 2020 Feb 5;15(2):e0228284. doi: 10.1371/journal.pone.0228284 (PMC7001916; doi:10.1371/journal.pone.0228284)

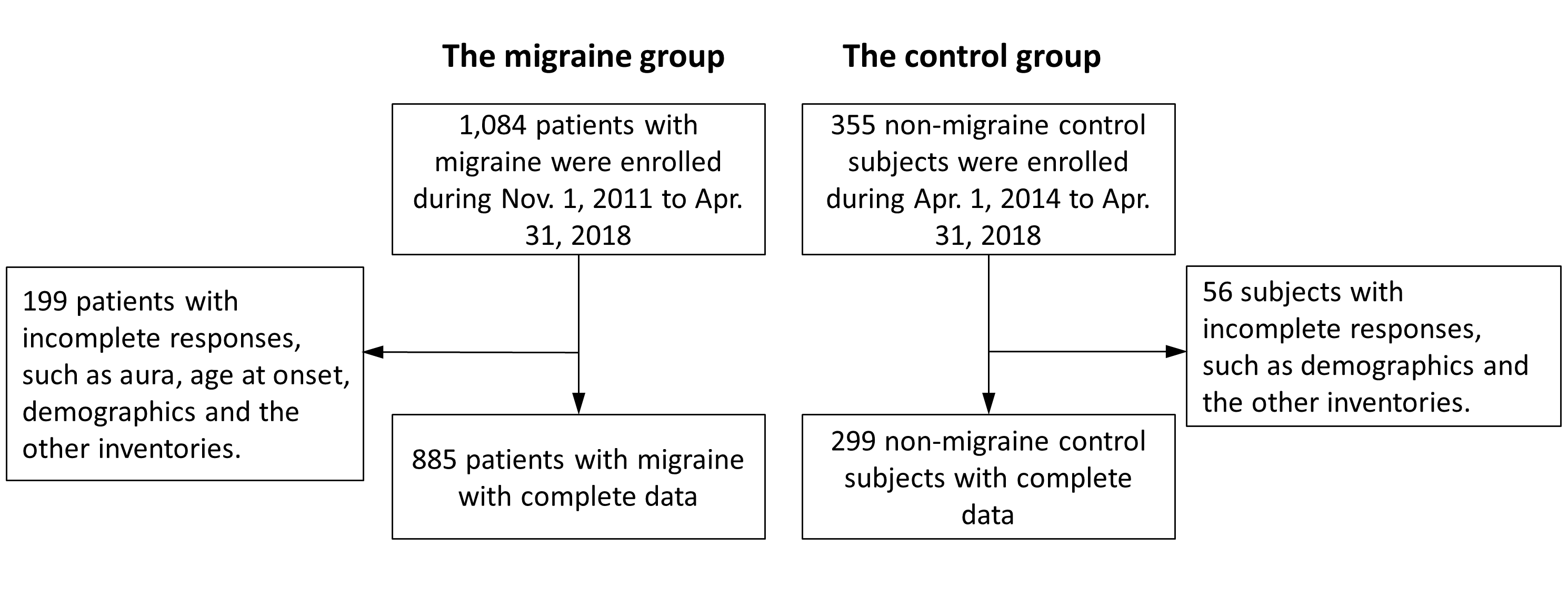

Supplement: S1 Fig — (TIF) [file pone.0228284.s001.tif]
